# ECG signal quality in intermittent long-term dry electrode recordings with controlled motion artifacts

**DOI:** 10.1038/s41598-024-56595-0

**Published:** 2024-04-17

**Authors:** Atte Joutsen, Alper Cömert, Emma Kaappa, Kirsi Vanhatalo, Jarno Riistama, Antti Vehkaoja, Hannu Eskola

**Affiliations:** 1 Faculty of Medicine and Health Technology, Tampere University, Tampere, Finland; 2 Finnish Cardiovascular Research Center, Tampere, Finland; 3 Department of Medical Physics, Tampere University Hospital, Tampere, Finland; 4 Faculty of Engineering and Natural Sciences, Tampere University, Tampere, Finland; 5 Royal Philips, Best, The Netherlands

**Keywords:** Biomedical electrodes, Electrocardiography, Materials testing, Signal-to-noise ratio, Skin–electrode interface, Impedance, Biomedical engineering, Sensors and biosensors, Cardiology

## Abstract

Wearable long-term monitoring applications are becoming more and more popular in both the consumer and the medical market. In wearable ECG monitoring, the data quality depends on the properties of the electrodes and on how they interface with the skin. Dry electrodes do not require any action from the user. They usually do not irritate the skin, and they provide sufficiently high-quality data for ECG monitoring purposes during low-intensity user activity. We investigated prospective motion artifact–resistant dry electrode materials for wearable ECG monitoring. The tested materials were (1) porous: conductive polymer, conductive silver fabric; and (2) solid: stainless steel, silver, and platinum. ECG was acquired from test subjects in a 10-min continuous settling test and in a 48-h intermittent long-term test. In the settling test, the electrodes were stationary, whereas both stationary and controlled motion artifact tests were included in the long-term test. The signal-to-noise ratio (SNR) was used as the figure of merit to quantify the results. Skin–electrode interface impedance was measured to quantify its effect on the ECG, as well as to leverage the dry electrode ECG amplifier design. The SNR of all electrode types increased during the settling test. In the long-term test, the SNR was generally elevated further. The introduction of electrode movement reduced the SNR markedly. Solid electrodes had a higher SNR and lower skin–electrode impedance than porous electrodes. In the stationary testing, stainless steel showed the highest SNR, followed by platinum, silver, conductive polymer, and conductive fabric. In the movement testing, the order was platinum, stainless steel, silver, conductive polymer, and conductive fabric.

## Introduction

The health care system is under pressure in the Western world due to an increasing life expectancy and a sedentary lifestyle, leading to growing numbers of chronic diseases. In the developing economies, people struggle to get access to health care. Both phenomena call for a dramatically different way of treating patients in the future, namely moving health care out of hospitals towards lower-cost, lower-personnel-intensive, and more accessible outpatient settings.

Cardiovascular diseases (CVD) are a major burden on society, as they cause the most deaths globally. An estimated 17.9 million people died due to CVDs in 2016, representing 31% of all deaths worldwide. Of these deaths, 85% are due to myocardial infarction and stroke^1^. The global annual cost of CVDs was approximately 863 billion USD in 2010, and it is estimated to rise by 22% to 1.044 billion USD by 2030, indicating that the burden of cardiovascular diseases will have a huge impact on the health care systems and the economy^2^. In the European Union, the total cost of cardiovascular disease is divided between 53% (111 billion EUR) in health care costs, 26% (€54 billion EUR) in productivity losses, and 21% (45 billion EUR) in the form of informal care for people with cardiovascular diseases^3^. In the United States cardiovascular spending increased from 212 billion USD in 1996 to 320 billion USD in 2016^4^. Recent national reports on the proportion of CVDs in the countries’ health care expenditure vary from 6 to 11.7%^5^. The United Nations has set 17 Sustainable Development Goals. Goal number three on good health and wellbeing includes the objective of reducing premature mortality from non-communicable diseases, including CVDs, through prevention and treatment by one third by the year 2030^6^.

A reliable diagnosis is the prerequisite for the effective treatment of any disease. In the case of CVDs, an electrocardiographic (ECG) recording reviewed by a trained medical professional is needed to diagnose the disease. The treatment process would also benefit from the monitoring of the patient’s status during the course of treatment to evaluate the response and to decide on adjustments. Cardiac arrhythmias are common^7^. They can be paroxysmal in nature, lasting from minutes to hours, or they can be persistent, where the arrhythmia lasts from days to months. Some arrhythmias can be asymptomatic, and the patient may be unaware of the condition. For example, 50%–87% of individuals with the most common type of arrhythmia, atrial fibrillation, are initially asymptomatic^8^. Reliable medical devices that can record clear and accurate ECG signals outside a clinical setting are needed to enable reliable diagnosis and effective treatment monitoring.

The gold standard method of studying the electrophysiology of the heart is the clinical 12-lead ECG, which gives a detailed snapshot of the current cardiac status. In addition to the short-term 12-lead recording, long-term recordings can be made using continuously recording ECG devices (Holter or patch devices) or implantable loop recorders. Intermittent ECG monitoring can be performed using hand-held devices. Additionally, the consumer market offers devices of varying form factors that are capable of recording ECG; however, only devices approved for medical use can be employed in clinical diagnosis and monitoring. All of the aforementioned devices require a fundamental element to interface with the signal source and the recording device: *the electrode*.

The electrodes can be divided into wet, dry, and capacitive electrodes. They can be separate from the device, built into the device, or integrated into a carrier such as a garment. The most common electrode type in clinical use is the disposable Ag/AgCl wet electrode. They are non-polarizable and stable, produce a high-quality signal, and are relatively resistant to motion artifacts, as they are fixed to the skin with an integrated adhesive and buffered by a gel pad^9^. Due to innovations in manufacturing, several companies produce them in masses, which leads to high cost-effectiveness in health care. However, they are not well-suited for long-term ECG monitoring, especially with ambulatory users. The gel tends to dry out, making the measurement prone to artifacts, and the commercially available electrodes feature a connector, which can disconnect in active ambulatory patients, causing loss of data^10^. Furthermore, the chemicals used in a wet electrode may cause contact dermatitis^11^. In clinical use, skin preparation—such as skin shaving, abrasion, and cleaning with a suitable oil-removing agent before electrode application—is usually recommended^12,13^. This may irritate the skin further. Preparation is more important in short-term (minutes, clinical 12-lead ECG) than in long-term recordings (days, Holter ECG), as skin impedance is reduced in minutes even with no skin preparation^14,15^. This is in accordance with the electrode–electrolyte interface model theory, which states that there exists a thin layer of tightly packed charge carriers at the interface from the electrode to the electrolyte^16^. The charge carriers in the electrolyte are ions and those in the electrode electrons. Therefore, charge carriers cannot migrate from one medium to the other, but a double layer will be created at the interface which acts as a communicating layer for the electric current. This layer will take some time to stabilize, after which it demonstrates a very constant electric behavior. However, every disturbance in the interface, such as electrode movement, will cause the double layer to lose its balance and force it to restabilize, showing transient electrical properties for a period of time. This causes both the interface noise and the contact impedance to fluctuate during the electrode material–dependent restabilization time. Furthermore, the effect of skin preparation may not markedly benefit long-term recordings, as complete skin regrowth occurs after 1–2 days^17^.

Capacitive electrodes feature an insulating layer between the electrode material and the skin. They are comfortable to wear, and they can be invisible to the user if they are integrated into a wearable device or garment. However, capacitive electrodes are also prone to motion artifacts^18^, which reduces their utility in ambulatory monitoring. Several research groups are developing capacitive electrodes, which may lead to unobtrusive and effective monitoring solutions in the future^19^.

The medical world is increasingly moving towards disposable devices due to patient safety and cost-efficiency, which feeds the growth of the medical disposables market^20^. The trend poses sustainability challenges. Sousa et al*.* have shown that reusable devices have a lower environmental impact compared to disposable devices^21^. In their Sustainable Development Goal number 12, the United Nations encourages responsible consumption and production^22^. Wearable cardiac monitoring with reusable dry electrodes that produce signals of sufficient quality may be beneficial in long-term monitoring applications while also being sustainable.

Dry electrodes have been used in research applications for a long time^14,23–26^, but they are not as established as wet electrodes. They have some drawbacks, such as high and varying skin–electrode impedance that depends on electrode location, skin properties, and electrode pressure^27^. In a recent publication, Kim et al*.* extensively reviewed the state of the art in dry electrode materials, covering their manufacturing methods, conductivity, signal quality, impedance, stretchability, breathability, and applications in wearable monitoring^28^. The rate of research publications on dry electrodes has increased exponentially from the start of the millennium due to advances in technology and new applications in wearable monitoring^29^. However, the literature in the field of dry electrodes lacks the knowledge of how dry electrode materials perform, particularly in long-term ECG monitoring with controlled motion artifacts. The purpose of this study was to test prospective motion artifact–resistant dry electrode materials for wearable ECG monitoring applications. The materials need to be biocompatible and robust and to endure cleaning. They must perform adequately without an added electrolyte, and they must not require the user to prepare either the electrodes or the skin in any way. The research questions in this study were: (1) What is the effect of various electrode materials on short- and long-term ECG signal quality? (2) What is the effect of electrode materials on motion artifacts in terms of short- and long-term ECG signal quality? (3) Are the various electrode materials tolerated without skin irritation in the long term?

## Materials and methods

**Figure 1:**
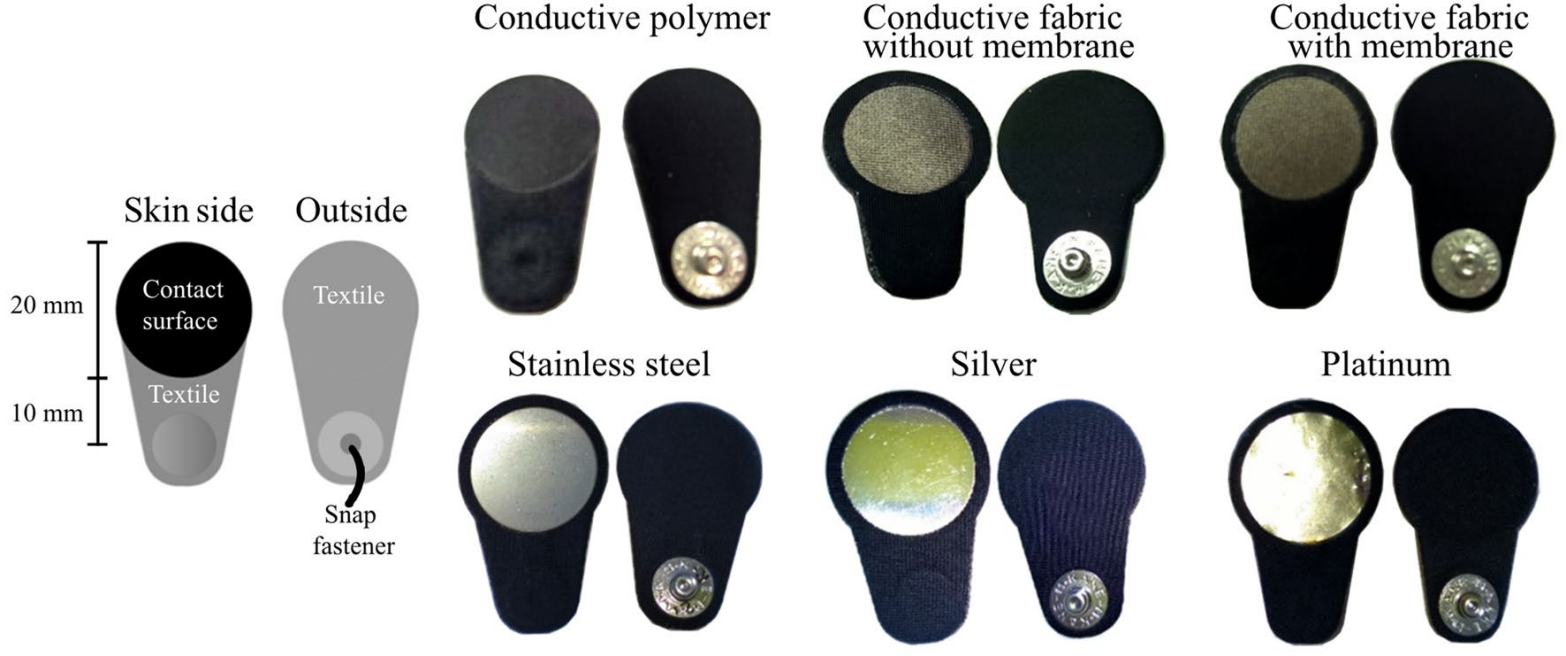
Six dry electrode types were manufactured for evaluation. The electrodes had identical contact surface areas and lead lengths, ending in a snap fastener enabling an easy connection to ECG recorder lead wires.

Five prospective motion artifact–resistant dry electrode surface materials that were biocompatible and have been used in biopotential recordings were selected: conductive polymer, conductive fabric, stainless steel, silver, and platinum (Fig. 1). Conductive polymers and fabrics are common in sports applications such as heart rate straps, stainless steel is often used as electrode material in gym equipment and wearables, silver is used in EEG cup electrodes, and platinum is used in implantable electrodes for stimulation and recording. The conductive polymer and fabric had a porous surface structure, while the stainless steel, silver, and platinum had a solid structure.

Six electrode types were manufactured from the five selected electrode materials. The conductive fabric was used in two electrode types, one with and one without an electrolytic moisture-retaining membrane. The membrane also functioned as an adhesive between material layers. All of the electrodes had a circular contact surface with a 20 mm diameter and a 10 mm lead ending in a snap fastener (Fig. 1).

The electrodes were manufactured by laser cutting the materials into shape and laminating them with surface textiles using heat-activated adhesive films (Table 1). Finally, silver-coated male snap fasteners were punched into place. Disposable gloves were used during the manufacturing and handling to avoid electrode surface contamination. The electrodes were stored in a dry and dark space in small, labeled plastic bags. Personal electrode sets were manufactured for each test subject to prevent cross-contamination.

**Table 1:** Electrode material layers from the outer to the skin-side surface.

Electrode type	Structure
Conductive polymer	Textile
	Adhesive membrane
	Polyurethane doped with carbon black
Conductive fabric without membrane	Textile
	Adhesive membrane, excluding the electrode contact area
	Silver-metalized spandex knit
Conductive fabric with membrane	Textile
	Adhesive membrane
	Silver-metalized spandex knit
Stainless steel	Textile
	Adhesive membrane
	SS AISI304 0.7 mm
Silver	Textile
	Adhesive membrane
	Ag (925) 0.3 mm
Platinum	Textile
	Adhesive membrane
	Flexible circuit board
	Polyester film 50 µm
	Copper foil 35 µm
	Gold 0.3 µm to improve electrolytic adhesion
	Electrolytic platinum 0.5 µm

The general cross-section of the arrangement was: textile—support materials—electrode surface material—skin.

**Figure 2:**
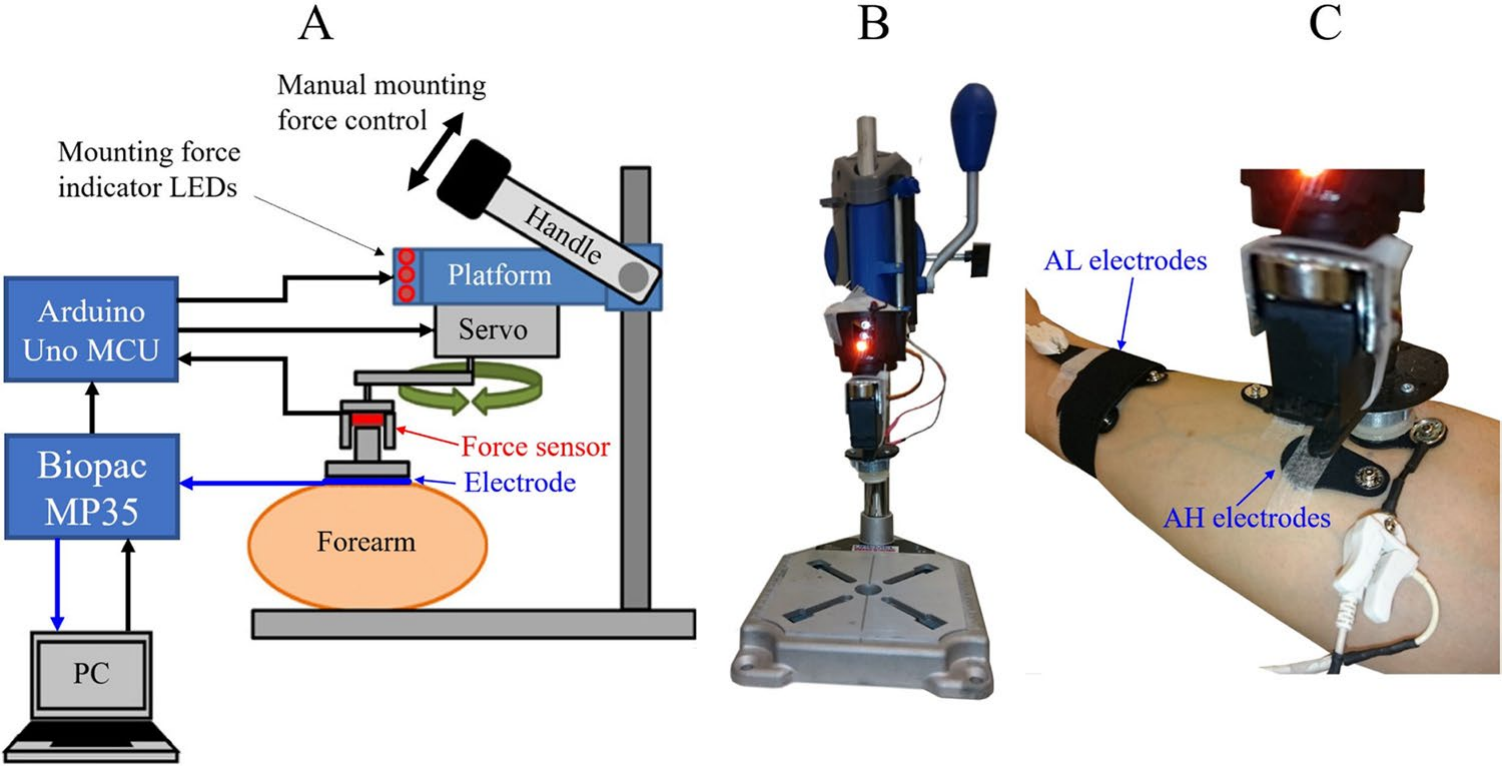
The instrumentation for producing the controlled mounting force and motion artifacts as well as for acquiring ECG recordings. (**A**) Schematic presentation and a lateral view of the instrumentation. Black arrows indicate commands and force sensor data, and blue arrows indicate ECG data. (**B**) Frontal view of the instrumentation. (**C**) The instrumentation’s servo and force sensor in place during electrode testing on a test subject’s medial forearm. AL and AH stand for the distal and proximal forearm electrode locations, respectively.

The manufactured electrodes were tested using a custom-made instrumentation to produce a controlled mounting force on the electrodes, to move the electrodes in a controlled manner to provoke motion artifacts, and to acquire an ECG recording (Fig. 2). Using such instrumentation ensures that the tests are standardized and repeatable between test subjects, and between tests on the same subject. The instrumentation has been developed and described earlier by Cömert and Hyttinen^30^ and successfully used in dry electrode motion artifact studies^26^.

The ECG recording was acquired using a Biopac MP35 device (Biopac systems Inc., CA, USA). The analog output of the Biopac was used to command an Arduino UNO microcontroller board (SmartProjects, Turin, Italy), which read the force sensor and controlled the mounting force indicator LEDs and the motion artifact servo movement. The mounting force was manually controlled by reading the LEDs and operating the handle (Fig. 2).

The ECG was recorded at a 200 Hz sample rate and a 0.05–100 Hz bandwidth. At the settings applied, the MP35 unit had a recording resolution of 3 μV. The differential mode input impedance was 2 MΩ and the common mode input impedance for AC was 1 GΩ. The servo movement frequency was set at 2 Hz and the amplitude at 4 mm. The 2 Hz frequency corresponded with foot impact frequency while jogging, and the 4 mm amplitude was large enough to cause motion artifacts but small enough to allow the skin to conform to the movement without causing the electrode to slide on the skin; see referred material for the instrumentation in operation^31,32^. The highest ECG signal quality and the fewest motion artifacts are recorded when the electrode–skin interface remains as stable as possible despite movement. To achieve this, the mounting force on the electrode should exert a pressure of between 15 and 20 mmHg on the skin below the electrode^33^. The pressure of textile-integrated electrodes on the skin was measured in 15 volunteers by placing a Microlab PicoPress M-700 sensor (Roncaglia di Ponte San Nicolò, Italy) under the electrodes of an elastic textile heart rate measuring chest strap with conductive polymer electrodes, adjusted to a comfortable but snug tightness. The median (min–max) pressure was 14 (7–36) mmHg. Relating the result to the test electrode surface area, a mounting force of 0.5–1.5 N was selected and programmed to the motion artifact device’s mounting force feedback LEDs. The LEDs had three pre-programmed indications: (1) too low force, < 0.5 N; (2) sufficient force, 0.5 N–1.5 N; and (3) too high force, > 1.5 N. The instrumentation had a hand lever that was used to press the instrumentation on the test electrode manually. The researcher operated the lever during the testing to light up the LED for indicating sufficient force for yielding an optimal electrode pressure on the skin.

Nine volunteer healthy adult test subjects, four women and five men, with no skin disorders in the intended test electrode skin locations were recruited. The median (min–max) anthropometric measures of the subjects were: age 38 (32–52) years; weight 76 (51–100) kg; height 176 (159–188) cm, and BMI 24.5 (20.2–28.3) kg/m^2^. The Prokerala and Fitzpatrick tests were used to evaluate the type and tone of the test subjects' skin^34–36^. The Prokerala test classifies skin into five different types: dry, normal, oily, combination (both oily and dry areas), and sensitive skin. The Fitzpatrick test categorizes skin based on its response to sunlight, ranging from type I (light) to type VI (dark). Dry and sensitive skin are more likely to develop dermal reactions, and higher-Fitzpatrick-score skin types are susceptible to pigmentary changes due to dermal abrasion. In the Prokerala test, five test subjects had normal skin and four had combination skin. In the Fitzpatrick test, six subjects had type III skin and three had type IV skin. For practical reasons, three to six electrode types were tested per subject and one to three electrode types were worn simultaneously. Each electrode type was thus worn by six test subjects.

**Figure 3:**
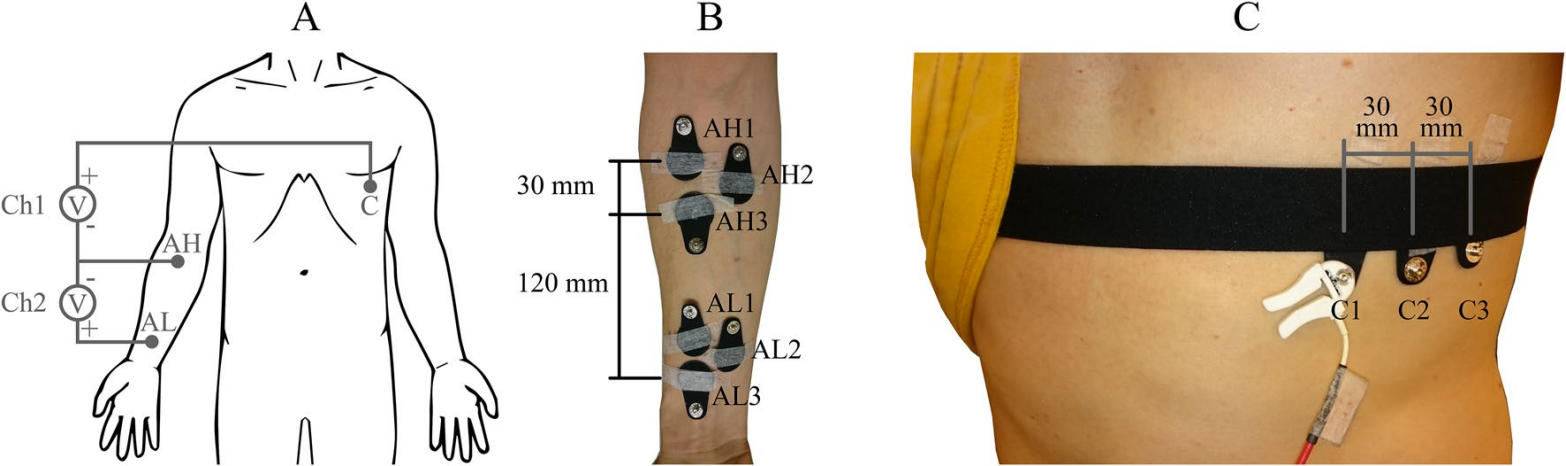
The electrode locations and recording channels. (**A**) Chest, Arm Low, and Arm High electrode positions, and the voltage channels Ch1 and Ch2 recorded from them. (**B**) and (**C**) electrode labels and inter-electrode distances. The electrodes in a set of three electrodes are of the same type. The electrode labels were C1, AH1, and AL1 in set 1; C2, AH2, and AL2 in set 2; and C3, AH3, and AL3 in set 3.

The electrodes were placed in three body locations: the thorax near the heart apex (chest, C), the right medial proximal forearm (arm high, AH), and the right medial distal forearm (arm low, AL) (Fig. 3). The electrodes were attached using medical skin tape. Elastic textile straps of the same type as was used in measuring electrode pressure were placed over the AL and C to ensure proper contact with the skin during the ECG recordings (Figs. 2C, 3C). For AH the skin tape was removed, and the electrode was mounted by the instrumentation (Fig. 2C).

After placing the electrodes, they were worn continuously for 48 h. The test subjects followed restrictions on exercise and skin care near the electrodes to avoid affecting the results. The trial started with a recording where the electrodes were allowed to settle onto the skin with the subject remaining stationary for ten minutes while continuous ECG was recorded. The trial continued with a recording where long-term intermittent ECG was recorded at 0 h (immediately after the settling test), at 24 h, and at 48 h. The recording at each time point consisted of a one-minute electrode movement provocation test, followed by a one-minute stationary test.

Data were acquired from two bipolar channels, Ch1 and Ch2. These channels had two individual electrodes (C and AL) and one shared electrode (AH) of the same electrode type (Fig. 3A). The mounting force on the individual electrodes was generated by elastic textile straps, and the shared electrode was mounted by the instrumentation. All electrodes remained still in the stationary tests, and in the movement provocation test, the shared electrode (AH) was moved by the instrumentation.

Signal-to-noise ratios (SNR) were calculated to compare the performance of the electrode materials. The QRS complexes in Ch1 were used to obtain the *signal* component, while Ch2 represented the *noise* component in the SNR calculation. Results were calculated for each minute in the settling test and for the middle 50 s of the one-minute movement provocation and stationary tests.

The frequency range spanning from 0.05 to 40 Hz contained 98% of the spectral power found within the entire recorded bandwidth, excluding technical artifacts originating from 10 and 50 Hz, along with their first harmonic frequencies. In an effort to mitigate the influence of low-frequency baseline fluctuations, the lower boundary frequency was adjusted from 0.05 to 0.5 Hz. As a consequence, the spectral power within the residual frequency band accounted for 70% of the total recorded spectrum. The SNR assessments were conducted in this 0.5 to 40 Hz frequency range. This selection was not designated for clinical application but rather for the purpose of eliminating technical disturbances, while retaining the majority of the ECG and motion artifact data.

The R-peaks in Ch1 were detected using a modified Pan-Tompkins method^30,37^. Similarly to the work described in^30^, the measure for the signal component was defined as the QRS amplitude, which was the range of the data in a 20-sample-long window corresponding to 100 ms around the detected R-peak. The measure for the noise component was defined as the corresponding Ch2 root-mean-square power of a 2-s window. Because low-amplitude QRS complexes were, in some cases, superimposed on the data in Ch2, a 100 ms segment around it was omitted from the noise calculation. The remaining 380 samples of the 2-s window were used for the calculation. Medians of both signal and noise metrics over the recording period were used for the SNR calculation (Eq. 1). The results were converted to decibels (dB) for visualization and tabulation (Eq. 2).

1 $$SNR=\frac{{signal}_{Ch1}}{{noise}_{Ch2}}=\frac{{med\left\{{max\left\{{QRS}_{i,j}\right\}}_{i=1}^{20}{-min\left\{{QRS}_{i,j}\right\}}_{i=1}^{20}\right\}}_{j}}{{med\left\{\sqrt{\frac{1}{380}\sum_{i=1}^{380}{(NOISE)}_{i,j}^{2}}\right\}}_{j}}$$

2 $${SNR}_{dB}=20 \cdot {{\text{log}}}_{10}\left(SNR\right)$$

**Figure 4:**
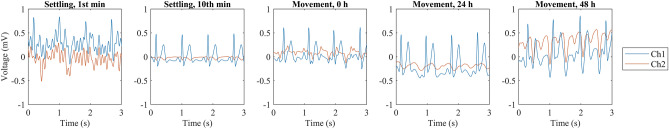
Example data from stainless steel test electrodes during the settling and long-term test. The figures illustrate the Ch1 (derivation C-AH, blue) and Ch2 (derivation AL-AH, red) after filtering. In the initial minute of the settling test, both channels exhibit noisy data, with only the R-peaks being discernible in Ch1. During the 10th and final minute of the settling test, the ECG waveform is distinctly visible in Ch1, whereas Ch2 appears as nearly isoelectric. Thereafter, one minute of movement provocation and stationary data were acquired intermittently at 0 h, 24 h, and 48 h. Here, only the movement test data are shown. The 2 Hz movement of the AH electrode and the resulting motion artifact are evident as periodic fluctuations in both channels. The motion artifact varies based on the properties of the skin and the skin–electrode interface at the time of the recording.

The R-peak amplitude is in the order of millivolts and the motion artifact in the order of hundreds of microvolts^30^. Therefore, it can be assumed that the time-varying motion artifact has a negligible effect on the numerator of Eq. (1) and that the numerator approximates pure signal component. The *med*, *max,* and *min* operators signify the median, maximum, and minimum values of the arguments. *QRS* signifies the segment spanning the QRS complex and *NOISE* the segment used to calculate the *noise* component. The index *i* is the 1–20 samples of the *QRS* segment and also the 1–380 samples in the *NOISE* segment. The index *j* is the 1–N segments in an individual test. For example, the N is 60 in a minute-long test with a heart rate of 60 beats per minute. In the movement provocation test, Ch1 was overwhelmed, in some cases, by the motion artifacts, which led to unreliable R-peak detection. In those cases, the *signal* component from the stationary test was used in place of the incomputable *signal* component of the movement provocation test. The quartile coefficient of distribution was calculated from linear-scale SNR results to quantify the spread of the results. Filtered signals from one test subject in settling and movement tests with stainless steel test electrodes are illustrated in Fig. 4.

Electrode–skin electric impedance was measured through the test electrodes during the ten-minute settling time, as well as during the movement provocation and stationary tests at 0 h, 24 h, and 48 h. The ECG and impedance measurements were made sequentially due to the limitations of the equipment. The impedance measurement was performed using a two-electrode configuration. An HF2IS impedance spectroscopy device (Zurich Instruments AG, Switzerland) was utilized to inject current and to measure the voltage drop between test electrodes in the AH and AL locations. Impedance data were acquired at seven distinct frequencies of injected current: 1 Hz, 10 Hz, 100 Hz, 1 kHz, 10 kHz, 100 kHz, and 1 MHz.

This method was used to measure the skin–electrode impedance of the electrode pair and the tissues below the electrode. The measurement lead impedances and connectors were included in the measurement but their impedances were negligible. The tissue beneath the test electrodes in the proximal forearm predominantly comprises skeletal muscle. Upon approximating the forearm as a cylinder with a radius of 4 cm, considering the resistivity of parallel muscle fibers to be 191 Ωm^38^, and with an inter-electrode distance of 12 cm, the resulting resistance was calculated to be 46 Ω. Therefore, the contribution of the tissue was also deemed inconsequential.

Single-electrode results are procured by dividing the measurement results by two, as the electrodes of the two-electrode setup were identical and the electrodes were connected in series in the electric circuit trough the skin. Impedance averages were computed for each minute and the frequency in the settling test, in addition to the movement and stationary tests. Impedance is a complex number having both magnitude and phase angle. For practical purposes, only the norm of the impedance vector is presented, as it influences the amplitude of the measured ECG signal.^39^.

**Figure 5:**
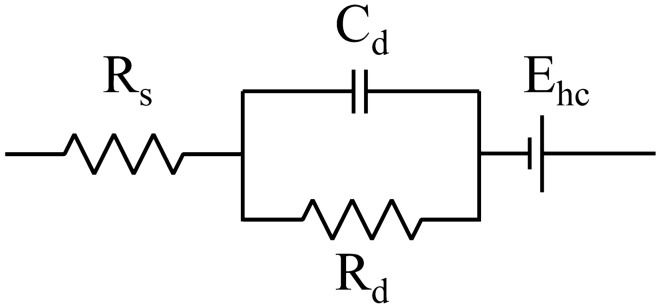
The single time constant model of the skin–electrode interface.

The recorded impedance norms over the 1 Hz–1 MHz band were fitted to the single time constant model of the skin–electrode interface impedance developed by Swanson and Webster^39^. The model has been used frequently to explain the impedance of the interface^40–44^. The model consists of a resistor R_s_ in series with a parallel connection of a capacitor C_d_ and a resistor R_d_, which is further in series with a voltage source E_hc_. R_s_ models the electrolyte and underlying tissue, C_d_ is the capacitance between the electrode and the skin, R_d_ is the resistance of the charge transfer between the skin and the electrode, and E_hc_ is the half-cell potential in the interface^41^ (Fig. 5). The impedance norm of this model is presented in Eq. (3). The fitting was executed by running a dense grid search of the component values and substituting them into Eq. (3). The component values were limited in that the resulting modeled impedance, a sigmoidal curve, passed through the measured impedance norm data points. The values that yielded the highest coefficient of determination, R^2^, between the measured data and the model were selected as the ones that best model the particular skin–electrode interface. Impedances across the entire 1 Hz–1 MHz band, as well as the average impedances within a 1–100 Hz sub-band that is relevant for most biosignals, are reported.

3 $$\left|Z\left(\omega \right)\right|=\left|Re\left(\omega \right)+jIm\left(\omega \right)\right|=\left|{R}_{s}+\frac{{R}_{d}}{1+{\omega }^{2}{C}_{d}^{2}{R}_{d}^{2}}-j\frac{\omega {C}_{d}{R}_{d}^{2}}{1+{\omega }^{2}{C}_{d}^{2}{R}_{d}^{2}}\right|=\sqrt{{\left({R}_{s}+\frac{{R}_{d}}{1+{\omega }^{2}{C}_{d}^{2}{R}_{d}^{2}}\right)}^{2}+{\left(\frac{\omega {C}_{d}{R}_{d}^{2}}{1+{\omega }^{2}{C}_{d}^{2}{R}_{d}^{2}}\right)}^{2}}$$

The test electrode noise was calculated from Ch2, where the two test electrodes were placed at a distance of 120 mm from each other on the medial side of the forearm and attached with an elastic band. The electrodes were left to settle for 5 min, followed by a 100-s noise recording.

The amplifier noise was measured by implementing the methods presented by Maji and Burke^45^. The amplifier inputs were connected through 100 Ω resistors to ground, and 100 s of noise was recorded with the amplifier’s highest sample rate, 2000 Hz. The amplifier noise was measured using a Biopac MP36 amplifier that, based on the manufacturer’s datasheets, had the same noise specifications as the MP35 used for the test electrode measurements.

The amplifier and test electrode peak-to-peak noise voltages were characterized by calculating the median of peak-to-peak voltages in one-second segments of the 100-s recording. The noise power spectral densities were calculated, summed, and multiplied with the frequency bin width to account for the frequency resolution. Both peak-to-peak noise voltage and total noise power were calculated from the recorded bandwidth of 0.05–100 Hz, excluding the common mode noise at 10 Hz and 50 Hz as well as their first harmonics. All signal processing was performed using Matlab (Natick, MA, USA).

### Ethical statement

The present material research does not constitute medical research as defined by Finnish law (9.4.1999/488), nor is it a clinical investigation as defined in Regulation (EU) 2017/745 of the European Parliament and of The Council. Therefore, an ethical review is not mandatory. The human subjects involved in the research were healthy adult volunteers recruited from the personnel of the participating organizations. All subjects gave their informed consent to participate in the research. The research procedures were in accordance with the general principles of the 1964 Declaration of Helsinki and its later revisions.

## Results

**Figure 6:**
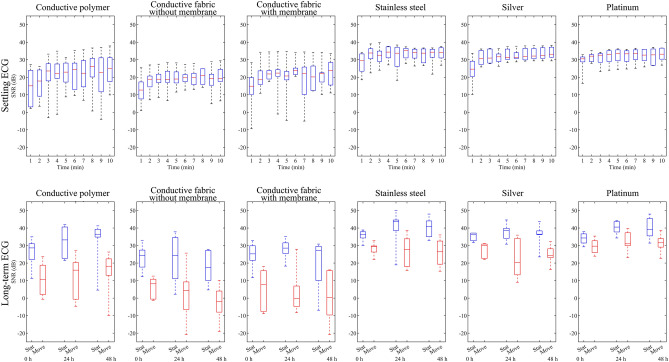
The ECG SNR in dB for all electrodes during the settling test (top row) and intermittent long-term tests (bottom row). The top and bottom borders of the box represent the first and third quartiles, respectively, and the line in the middle is the median. The whiskers indicate the highest and the lowest values. In the long-term test, the blue boxes show the result for the stationary and the red boxes for the movement provocation test.

The results of the SNR calculations are illustrated in Fig. 6. A higher SNR signifies better performance. In the 10-min settling test, the recording was started when the electrodes made skin contact. The SNRs of all electrodes show an upward trend. The SNR first increases rapidly and then plateaus after approximately 3–5 min. The SNRs of the stainless steel, silver, and platinum electrodes are higher and show less variation than those of the conductive polymer and fabric electrodes.

In the long-term stationary test, the SNRs are elevated from the initial 0 h recording to the 24 h and 48 h recordings, except for the conductive fabric electrode without a moisture retaining membrane. The stainless steel, silver, and platinum electrodes show higher SNRs than the conductive polymer and fabric, similarly to in the settling test (Fig. 6).

In the long-term controlled movement provocation test, the electrodes were oscillated from their stationary position, causing a motion artifact voltage superimposed on the ECG waveform. The SNRs dropped markedly from the stationary test. The stainless steel, silver, and platinum electrodes show higher SNRs than the conductive polymer and fabric electrodes, as in the long-term stationary ECG. The conductive polymer shows an upward trend in both the stationary and the movement provocation tests, whereas the conductive fabric without a membrane has a downward trend for both tests in the long-term ECG evaluation. The other electrodes show no clear visual trend (Fig. 6).

The numerical SNR results of the intermittent long-term tests and the quartile coefficients of dispersion over all of the results are summarized in Table 2. In general, the electrodes’ SNRs are elevated with longer recording times. In the movement provocation test, the stainless steel, silver, and platinum electrode materials show overall higher SNRs than conductive polymer and fabric, whose SNR is reduced to a fraction of the stationary test. The conductive fabric electrode with a moisture-retaining membrane shows slightly higher SNRs than the electrode without the membrane. This advantage is lost during the movement provocation. Stainless steel has the highest SNR in the stationary recordings, followed by platinum, silver, conductive polymer, and conductive fabrics. In the movement provocation test, platinum, and stainless steel switch places, while the remaining materials retain the same order. The quartile coefficient of dispersion was found to be higher in porous than in solid electrodes. All of the calculated SNR and impedance results, as well as a video on the instrumentation applied in movement provocation during ECG and impedance recordings, are available online at IEEE DataPort^31^ and Mendeley Data^32^.

**Table 2:** The overall numerical SNR results in dB of the intermittent long-term ECG testing.

Electrode surface material	Signal-to-noise ratio medians (dB)						Quartile coefficient of dispersion over all results
	0 h, stationary	24 h, stationary	48 h, stationary	0 h, movement	24 h, movement	48 h, movement	
Conductive polymer	28.7	33.2	36.5	10.7	16.0	18.2	0.66
Conductive fabric without membrane	24.3	24.4	17.4	8.2	4.4	− 1.9	0.61
Conductive fabric with membrane	25.3	28.5	27.2	7.8	-0.3	0.3	0.68
Stainless steel	36.2	43.8	40.9	29.4	27.7	26.5	0.41
Silver	36.2	38.5	36.5	30.0	20.4	24.2	0.39
Platinum	34.3	40.5	39.0	29.7	31.0	31.6	0.35

The results are medians from the respective time points. The quartile coefficient of dispersion portrays the spread of the results produced from settling and long-term testing.

**Figure 7:**
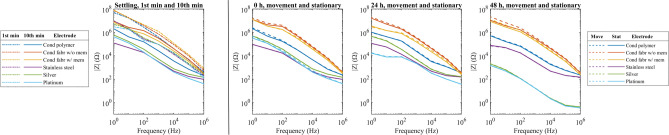
Impedance data over the recorded 1 Hz–1 MHz band from all of the test electrodes during the settling and long-term test. The far left panel depicts 1st- and 10th-minute results of the settling test. The following three panels show the intermittent long-term movement provocation and stationary test results. Overall, the solid materials consistently exhibit impedances that are lower than those observed in porous materials.

The skin–electrode impedance results are presented in Fig. 7. In the continuous 10-min settling test involving impedance recording, the observed results reveal elevated low-frequency impedances compared to high-frequency impedances over the recorded 1 Hz–1 MHz band. Throughout the settling test, there was a consistent reduction in all impedance values. Specifically, the solid electrodes exhibited an average impedance reduction of approximately 88%, while the porous electrodes experienced a reduction of roughly 77% over the 10-min period. The solid electrode materials consistently maintained impedances of approximately one order of magnitude lower than the porous electrodes across the recorded frequency range.

Many biosignals manifest predominantly in the low-frequency spectrum. Focusing on the 1–100 Hz range, the initial impedance for porous electrodes was approximately 30 MΩ, while solid electrodes demonstrated an impedance of around 3 MΩ within the first minute of the settling test. Towards the final minute, these impedances stabilized as close to 3 MΩ and 0.2 MΩ for porous and solid electrodes, respectively.

In specific electrode materials, the conductive polymer of the porous materials and the platinum of the solid materials exhibited the lowest impedances averaged over the recorded 1 Hz–1 MHz band during the first minute of the settling test. After the 10 min, the conductive polymer maintained its position as the porous electrode with the lowest impedance, whereas stainless steel emerged as the solid electrode material with the least impedance.

In intermittent recordings at 0 h, 24 h, and 48 h, porous materials showed an average of 14% and solid materials 4% lower impedances during stationary conditions than during motion provocation tests. Focusing on the 1–100 Hz biosignal band over the 48-h period, the movement provocation test impedance values of porous materials spanned the range of 4–5 MΩ. Solid electrodes exhibited a continuous decrease in impedance from 200 to 20 kΩ. Similar trends were noted in the intermittent stationary tests, where porous electrodes showed impedances ranging from 3 to 4 MΩ, while solid electrodes experience a reduction from 100 to 20 kΩ over the 48 h.

In the intermittent testing, conductive polymer electrodes demonstrated the lowest average impedances in both movement and stationary tests among the porous materials over the 1 Hz–1 MHz band. Among solid materials, stainless steel exhibited the lowest overall impedances during the 0-h motion and stationary tests; however, in the subsequent 24-h and 48-h tests, platinum emerged as the material with the lowest impedance.

**Figure 8:**
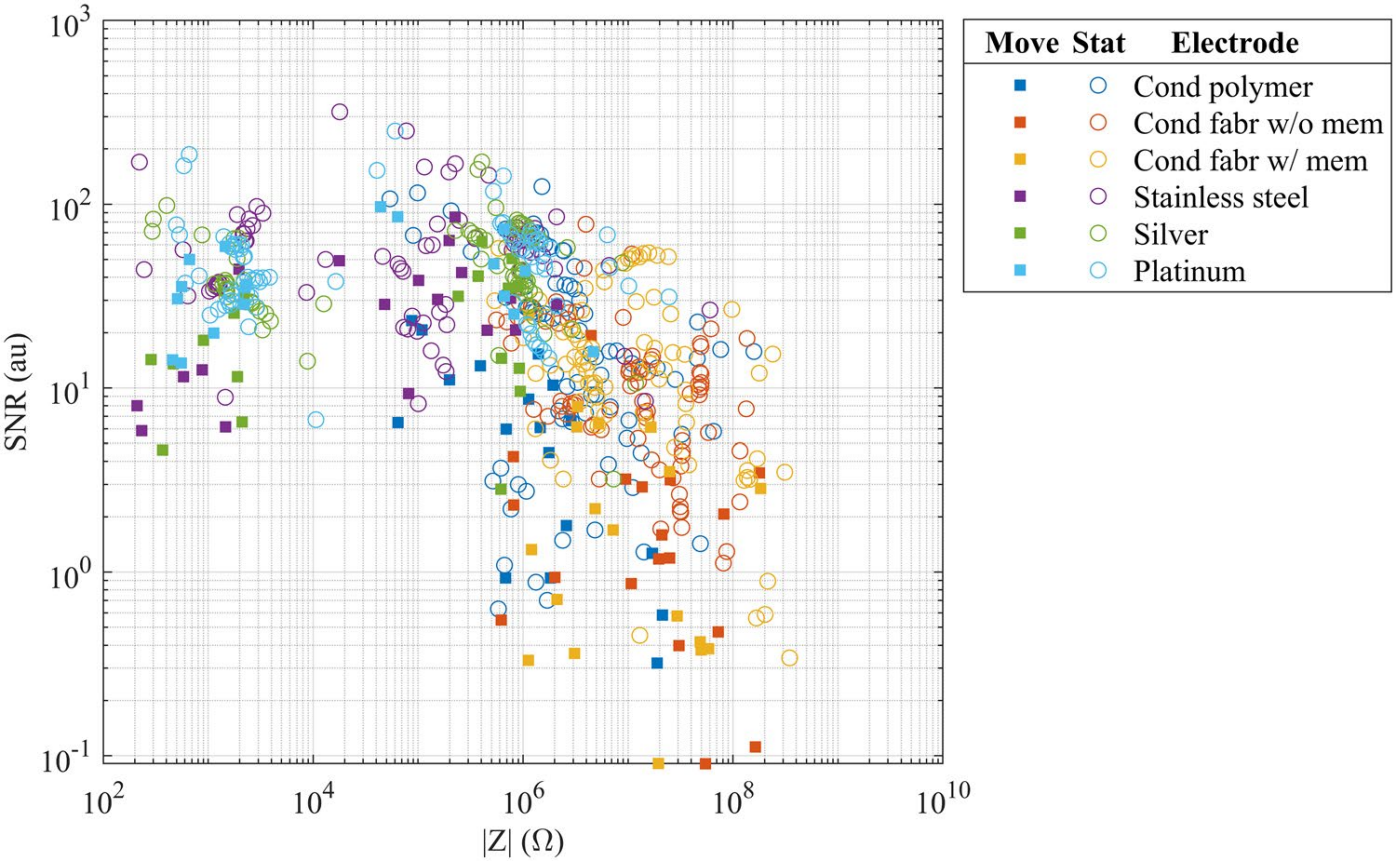
Test electrode’s skin–electrode interface impedance in the 1–100 Hz biosignal band and SNR from the movement and stationary tests. The stationary data include average impedances from each minute of the settling test, as well as the one-minute tests at 0 h, 24 h, and 48 h. The movement data contain average impedances in the minute-long movement provocation testes at 0 h, 24 h, and 48 h.

The relationship of skin–electrode interface impedance in the 1–100 Hz biosignal band and SNR is illustrated in Fig. 8. Generally, electrodes featuring porous surface materials demonstrate elevated skin–electrode impedance and lower SNR when compared to electrodes with a solid surface. The loglog plot indicates a negative relationship between impedance and SNR. Spearman’s rank correlation coefficient showed an association of ρ = − 0.61 between the skin–electrode interface impedance and the SNR in static testing and ρ = − 0.64 in movement testing. Both results indicate a moderate to strong negative association between impedance and the SNR.

The skin–electrode interface was modeled using the single time constant model. The model was fitted to the recorded impedance data from the 10th minute of the settling test when the skin–electrode interface had stabilized. The model fit the recorded impedances well (R^2^ > 0.9) with the found R_s_, R_d_, and C_d_ values. The data fitting was inspected visually to verify successful fitting. The porous materials showed higher resistor and lower capacitor component values than the solid materials. Conductive polymer and platinum had the lowest resistor values and highest capacitor values of the porous and solid materials, respectively (Table 3).

**Table 3:** Medians of the modeled component values of the skin–electrode interface, R_s_ + (R_d_ || C_d_). Values were modeled based on the 10th-minute settling impedances.

Electrode	R_s_ (Ω)	R_d_ (MΩ)	C_d_ (nF)
Conductive polymer	220	2.02	4.64
Conductive fabric without membrane	249	8.94	0.440
Conductive fabric with membrane	226	7.38	0.675
Stainless steel	102	0.122	60.5
Silver	167	0.593	59.3
Platinum	48	0.418	5440

**Figure 9:**
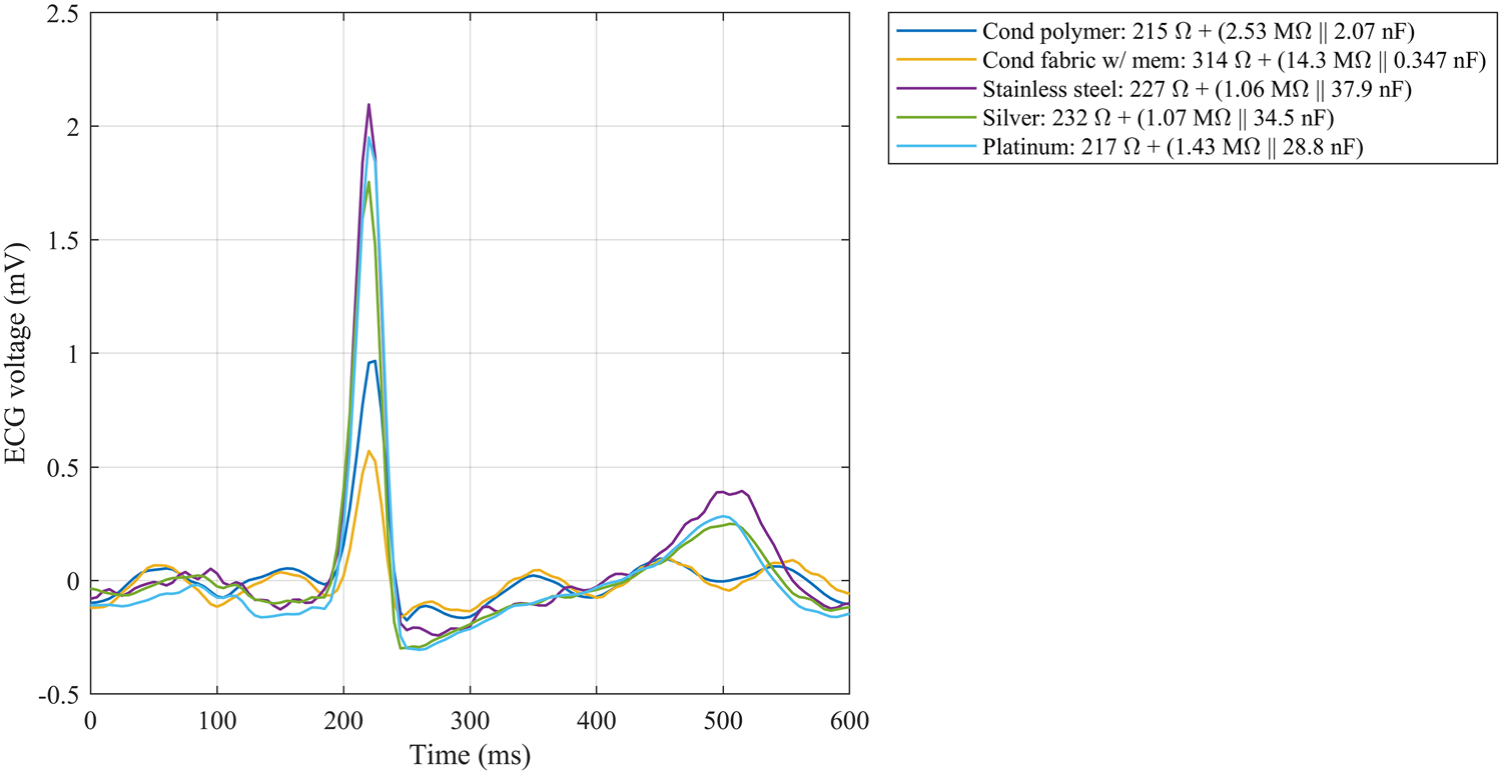
ECG over one cardiac cycle from a test subject wearing five test electrodes, one of each surface material. The data have been recorded during the 10th minute of the settling test. Due to limitations of the recording equipment, the data were recorded sequentially and processed and aligned in post-processing. Some residual, 10 Hz common-mode interference is seen in the porous electrode data. The skin–electrode interface model component values for the presented case are in the figure legend.

ECG waveforms recorded using five test electrode types are illustrated in Fig. 9.The waveforms are all from the same test subject to enable comparison. The ECG amplitudes vary depending on the impedance of the skin–electrode interface. Higher impedances lead to lower ECG amplitudes. The electrodes with a solid material displayed a higher amplitude ECG than the electrodes with porous materials, as R_s_ || C_d_ yield lower impedances because of lower R_d_ and higher C_d_ component values. The differential input impedance of the amplifier, 2 MΩ, coupled with the high-impedance dry electrodes led to signal undershoot and fast recovery after the R-peak (Fig. 9). The undershoot and recovery slopes are listed in Table 4.

**Table 4:** Undershoot and recovery slope after R-peak calculated from Fig. 9.

Electrode	Undershoot (µV)	Recovery slope (µV/s)
Conductive polymer	175	1000
Conductive fabric with membrane	163	905
Stainless steel	242	1790
Silver	298	1660
Platinum	305	1910

The results of the peak-to-peak noise voltage and total noise power calculations of the bipolar measurement are presented in Table 5. The amplifier noise voltage was 1.7–4.2% of the combined amplifier and electrode noise voltage and the amplifier noise power 0.5–2.5‰ of the total noise power. The results were divided into three groups based on their measured noise voltages. Solid electrodes showed the lowest noise voltages, followed by conductive fabric electrodes. The conductive polymer electrode had the highest noise voltages, approximately double that of the solid electrodes. In the total noise power, the metallic electrodes showed low noise and the conductive polymer exhibited four times as much noise power.

**Table 5:** The peak-to-peak noise voltage and total noise power of the bipolar channel over the recorded 0.05–100 Hz recorded band.

Electrode	Noise V_p-p_ · 10^–6^	Total noise power V^2^/Hz · 10^–9^
Conductive polymer	408	39.3
Conductive fabric without membrane	264	11.7
Conductive fabric with membrane^a^	265	8.17
Stainless steel	166	11.5
Silver	197	8.04
Platinum	219	13.7
Biopac MP36 amplifier	6.98	0.0198

^a^Due to artifacts in the data, the results for the conductive fabric with the membrane electrode were not calculated after 5 min of settling but rather after 4 min.

The test subjects were interviewed after the 48-h exposure to the electrodes. None of them reported irritation caused by any of the electrodes. The skin under the electrode sites was also visually inspected after removing the electrodes. No skin irritation was observed in any of the subjects. The skin under the solid electrodes was moister and flatter, and had a more clear electrode shaped indentation than under the porous electrodes. The only slight erythema that was found was on skin that had been under the medical adhesive tape.

## Discussion

Five dry electrode surface materials were tested in six electrode structures. The electrode materials remained in continuous contact with the skin for 48 h. Intermittent ECG was acquired three times during the 48-h study period. Signal-to-noise ratios were used to quantify the quality of the ECG signals. SNR calculations have been used in the literature to study ECG signals after recording or signal processing. The SNR calculation methods have employed power ratios without segmenting the signal^46–50^, squared and summed signal-to-noise ratios^51^, as well as signal-to-noise estimation of segmented signals^52^, or they have been undescribed^53,54^. Cömert has shown that textile electrodes have a low SNR, limiting the analysis of the lower-amplitude components, the P and T wave, of the ECG^33^. Many cardiac metrics, such as the R-R interval, heart rate, and heart rate variability, are based on identifying the ventricular contraction phase from the ECG. Therefore, the prominent QRS complex from the chest–forearm channel (Ch1) was used for the signal component and the simultaneous data from the forearm channel (Ch2) for the noise component in the SNR calculation, similarly to the work by Cömert and Hyttinen^30^.

### Skin–electrode area with solid and porous materials

The six electrode structures are clearly divided into two groups based on the SNR results. The solid materials (stainless steel, silver, and platinum) showed higher SNRs in all the tests when compared with porous materials (conductive polymer and fabric). A larger skin–electrode interface area translates into a higher SNR^25,55^. The solid materials have their whole surface area pressed to the skin. The materials with some roughness to their surface structure may lose contact area because only the “peaks” of the material are in contact with the skin and not the “valleys”. This can be compensated for by using an electrolyte that fills the valleys. Wu et al*.* compared textile dry electrodes with different conductive knit patterns on test subjects. They found that jacquard-pattern plain textile electrodes have a lower skin–electrode impedance and a higher ECG SNR compared to a single jersey honeycomb pattern, which has repeating concave microstructures in its topography that do not contact the skin^56^. Huigen et al*.* studied the noise sources in hydrogel and wet-gel electrodes. They discussed the behavior of these electrolytes in the skin–electrode interface and suggested that the wet-gel, a loose flowing gel usually contained in a sponge, fills the skin’s inhomogeneities better and results in an increased contact area in comparison to a solid adhesive hydrogel^57^. However, conductive gel is not a viable option in long-term wearable measurement applications. The natural moisture, i.e. sweat containing Na+, Cl− and K+ ions^58^, that accumulates in the skin–electrode interface forms a conductive path between the skin and the electrode^24^ and may increase effective surface area by at least partially filling the valleys of the electrode material. The impedance of the skin’s stratum corneum is lowered over time as the dry outer layer of the skin is moisturized^15^. Moisture also stabilizes the half-cell potential as it buffers the movement of the electrode relative to the skin. The moisture may also help to attach the electrode surface to the skin, thereby further stabilizing the skin–electrode interface. Without the moisture acting as electrolyte, the electrode is working more in a capacitive mode, as the stratum corneum layer acts as an insulator, making the measurement prone to motion artifacts. It may be that, in the present study, the moisture was not able to increase the porous electrodes’ effective surface area. Moisture may escape from porous materials through the material and from the edges of the material, elevating the impedance of the skin–electrode interface, promoting artifacts and leading to a low SNR. The smooth, solid materials retain the moisture better, as it is only allowed to evaporate from the edges of the electrode. When the electrodes were removed after the 48 h, the skin was observed to be moister and flatter than the surrounding skin and to have an indentation in the shape of the electrode. This effect was more pronounced with the solid electrodes.

### Settling and long-term test SNR

In the settling test, all test electrodes showed a rising SNR trend. The electrode’s coupling with the skin is improved as the natural humidity evaporating from the skin is captured in the skin–electrode interface. The conductive fabric electrode without the membrane backing allows the moisture to escape and therefore shows the lowest SNR trend of the six electrode structures. Searle and Kirkup measured the impedances of dry electrode pairs and found that the impedance decreases exponentially and levels out after about 200 s^14^. The settling SNR results of the present study reveal a similar but inverted gradient and levelling. In the long-term test, the electrodes were subjected to both movement provocation and stationary conditions. The servo movement simulated the movement of a wearable electrode in contact with the skin during jogging. All of the electrodes’ SNRs were markedly reduced in the movement provocation test compared to the stationary test.

In stationary conditions, the SNRs of all of the metallic electrodes peaked at 24 h and declined slightly towards 48 h. The SNR of the conductive polymer continued to rise throughout the long-term test in both stationary and mobile conditions. In our unpublished case study, we discovered that, when recording heart rate for up to 7 days using an elastic textile chest strap with integrated conductive polymer electrodes, the electrodes adhered to the skin very tightly, which improved the robustness of the measurement. On the other hand, prolonged use of a conductive polymer electrode was noticed to cause skin irritation. In the unpublished study, the electrode surface area was larger and the exposure longer than in the present study, which have may influenced how the skin under the electrodes reacted.

The results presented in Fig. 6 show high spread of the SNR. The quartile coefficient of dispersion is a robust method to quantify the spread of data. The results show that the porous electrodes have a higher dispersion of 0.61–0.68, whereas the solid electrodes have a dispersion of 0.35–0.41. The even surface structure of the solid electrodes may compensate for the micro-scale unevenness of the skin and retain moisture better, leading to a more stable skin–electrode interface, higher SNRs, and a lower spread of results compared to the porous electrodes.

### Silver electrodes

Three electrode types had silver as the electrode surface material. The solid silver outperformed the porous conductive silver fabric electrodes. Therefore, it appears that surface solidity, which may lead to better electrolytic moisture build-up, is a more important property than surface material alone. Otherwise, the silver-coated conductive fabrics would have performed similarly to the solid silver electrode.

The two conductive silver fabric electrode structures were different in terms of how they allowed the moisture to escape from the skin–electrode interface. The structure with the moisture-retaining membrane performed slightly better than the structure without the membrane in the stationary test. However, the advantage was lost in the movement provocation test. The conductive fabric electrodes showed the lowest SNRs. The moisture-retaining property of the electrode with the membrane did not translate into a substantially higher SNR than in the electrode without the membrane during the movement provocation test. Therefore, electrode surface porosity, rather than the use of a membrane, seemed to dictate the overall results for the conductive silver fabric electrodes.

### Pressure

The SNR of the electrode is also related to the pressure of the electrode on the skin. A loosely bound electrode is prone to artifacts, as it easily moves relative to the skin. Cömert et al*.* found while using textile electrodes and varying mounting forces that, for optimal signal quality, the pressure under the electrode should be 15–20 mmHg when recording ECG^33^. Li et al*.* found that a pressure of 1297 ± 102 Pa (9.728 ± 0.765 mmHg) in an upper arm band with screen-printed dry electrodes yields the highest SNR in ECG^59^. Takeshita et al*.* report that contact pressures below 1000 Pa (7.501 mmHg) lead to noisy signals with baseline drifting in textile-integrated ECG electrodes^60^. However, it should be noted that too high a pressure leads to decreased signal quality as well as reduced wearing comfort of the wearable device^33,59^. In the present study, the pressure under textile-integrated electrodes in a heart rate chest strap adjusted to a comfortable but snug tightness was measured. The median result, 14 mmHg, was slightly below what Cömert et al. recommend but above the finding of Li et al. The range of the pressure measurement results, 7–36 mmHg, covers the range of both studies. Based on these, an appropriate mounting force of 0.5–1.5 N was selected to ensure that the SNR is optimized regarding the pressure within the skin–electrode interface.

### Impedance

The skin–electrode impedances of test electrode pairs were recorded by injecting currents at seven specific frequencies in a 1 Hz and 1 MHz band and by measuring the voltage drops during the tests. It should be noted that the SNR was calculated from the 0.5–40 Hz band, whereas the skin–electrode impedances in Fig. 7 are from the 1–100 Hz band. This was due to the limitation of the study design. It is unlikely that the mismatch of the analysis bands would affect the conclusions drawn from the results, as the band used for the ECG SNR analysis contained 70% of the spectral power of the full band. Therefore, most of the information used for calculating the SNR was retained.

The correlation of the skin–electrode interface impedance and SNR was studied by visualizing and calculating the relationship between skin–electrode impedance in the 1–100 Hz biosignal band and SNR. Both the visualization and Spearman’s rank correlation coefficient showed that SNR tends to decrease with increasing impedance. Spearman’s ρ negative association was moderate to strong. Spearman’s rank correlation coefficient does not necessarily indicate a linear relationship, but rather a monotonic one, meaning that when there is an elevation in Z, there tends to be a decline in the SNR. The Z and SNR associations in the stationary and movement test were ρ = − 0.61 and ρ = − 0.64, respectively. The difference may be attributed to fewer datapoints in the movement test and dispersion in the data, rather than a significant difference between the association results.

Almasi and Schmitt showed that ECG can be distorted due to suboptimal skin–electrode interface impedance. To remedy the problem, they suggested decreasing the skin–electrode interface impedance and increasing the input resistance of the amplifier^61^. Taji et al*.* modeled the skin–electrode interface electric components based on impedance measurements. They calculated the undistorted in-body ECG from modeled impedance and ECG measured from textile electrodes. The impedance is frequency-dependent, and it was high in the band below 20 Hz and caused ECG signal attenuation^62^. Goyal et al. researched titanium and stainless steel dry electrodes and found that high skin-electrode impedance resulted in a lowered ECG SNR. The effect was more pronounced with individuals with dry skin than with hydrated skin^63^. The results of the present study concerning the association of impedance and signal quality are in line with the literature. Therefore, electrode design should be focused on achieving low skin–electrode interface impedance to gain a high signal quality.

In the reviewed literature, the dry electrode skin–electrode interface component values are 16.7 Ω–100 kΩ for R_s_, 78.8 kΩ–4 MΩ for R_d_, and 7–52.7 nF for C_d_^41,42,44,64–67^. The modeled component values in the current work are in the same range, except for the conductive fabric electrodes that showed a higher R_d_ and lower C_d_, possibly due to the lack of an electrolyte in the skin–electrode interface and a reduced effective contact area due to the material porosity.

The solid electrode materials paired exceptionally well with the skin of test subjects number 8 and 9, causing low impedances that reflected particularly the high platinum electrode C_d_ component value (Table 3). This effect of low skin–electrode impedance may be attributed to individual variance in the skin’s electric properties^17,61,68,69^.

Modeling the skin–electrode impedance to determine its electrical component values is important because it dictates the input resistance of the biopotential amplifier. Maji and Burke have investigated the amplifier specifications related to fulfilling the IEC 60601-2-25 standard requirements for the essential performance of electrocardiographs. To avoid signal distortion, the standard states that the system’s response to a 100 ms and 3 mV rectangular pulse must not exceed the undershoot limit of 100 µV and the recovery slope limit of 300 µV/s^70^. The results of simulations and tests by Maji and Burke suggest that an amplifier input impedance exceeding 3 GΩ is needed to avoid distortion of the ECG signal using dry electrodes, equating an R_d_ of as high as 4 MΩ and a C_d_ of as low as 7 nF^40^. Considering the fact that the conductive fabric test electrodes in the present study showed an R_d_ of 7 MΩ and 9 MΩ and a C_d_ of 0.4 and 0.7 nF, the input impedance requirement is even greater, up to 7 GΩ, as extrapolated from the results of Maji and Burke. The Biopac MP35 data acquisition unit has a differential input impedance of 2 MΩ only, which is clearly too low for dry electrode testing. In Fig. 9, the signal undershoot after the R-peak violates the standard’s limit of 100 µV and the recovery slope limit of 300 uV/s in all cases. However, it should be noted that part of the undershoot may be actual S-wave. As the ground truth ECG is unknow here, there are uncertainties to the results in the presented undershoot and recovery slopes in Table 4. Regardless, amplifier input impedance should be considered when recording dry-electrode ECG to avoid the risk of misinterpreting pronounced signal distortion after the R-peak as pathological ST depression caused by myocardial ischemia^40,71^.

The latest revision of the IEC 60601-2-25 standard is from 2011. Therein, the test circuit skin impedance equivalent components are a 51 kΩ resistor in parallel with the 47 nF capacitor. These values are in the range of wet Ag/AgCl electrodes^43,44,64,65,72^, and, therefore, test specifications need to be added for dry electrodes in future revisions.

In the literature, analyses of the skin–electrode interface impedance for a bipolar channel typically assume that the electrodes have equivalent impedances^40–42,44^. In reality, the skin–electrode interface may have unequal impedances due to local skin properties, the amount of electrolyte, and the pressure of the electrode on the skin. The higher the impedance, the more likely it is that the impedances are further apart. When the common-mode signal passes through the mismatched impedances, it is converted into a differential signal. This is due to the potential divider effect formed by the skin–electrode impedance and the amplifier input impedance^73^. When the electrode impedances are mismatched, the input signal is seen as differential-mode instead of common-mode. In case of impedance mismatch, the CMRR will be degraded as approximately ΔZ_E_/Z_C_, where ΔZ_E_ is the difference between the two skin–electrode interface impedances and Z_C_ is the input impedance of the operational amplifier. The result of electrode impedance mismatch is a reduced common-mode rejection ratio of the amplifier and ECG signal with some coupled common-mode signal, usually 50/60 Hz mains interference. This potential divider effect may be the major CMRR-lowering factor in dry electrode recordings with high and imbalanced impedances^74^. In the present study, the raw data were contaminated by common-mode signals at 10 Hz and 50 Hz, which was identified as sharp peaks in the power spectrum.

Amplifiers have DC bias currents that flow through the skin–electrode interfaces and produce DC potentials. When CMOS- or JFET-based amplifiers are selected, the currents are of pA magnitude. Even with high impedances of tens of megaohms, the resulting voltages are very low, in the 10 µV scale, compared to electrode offset voltages that can be in the hundred-mV scale^74^. Furthermore, in ECG recording, the DC and very low baseline drifts are filtered out to avoid saturating the amplifier and complicating the interpretation^40,75^.

### Comparisons to dry electrode studies with movement provocations

Searle et al*.* experimented with a stainless steel dry electrode, insulating electrode, and wet electrode to find their motion artifact magnitude during controlled movement provocation of over 15 min. At the beginning of the test, the artifact voltages (V_RMS_) of stainless steel and insulating electrodes were markedly higher than those of the wet electrode. In the end, the wet electrode’s V_RMS_ was nearly the same, but the V_RMS_ of stainless steel and insulating electrodes had decreased to well below that of the wet electrode. At the end of the recording, stainless steel showed the lowest V_RMS_^14^. Bergey et al*.* compared silver, gold, brass, stainless steel, and anodized aluminum. They found that stainless steel showed the fewest motion artifacts^23^. Gruetzmann et al*.* tested a silver dry electrode, two types of conductive foam electrodes with silver coating, and an Ag/AgCl gel electrode in a walking test. The foam electrodes demonstrated the fewest motion artifacts, followed by the silver dry electrode and the Ag/AgCl gel electrode^24^. Meziane et al*.* compared one gel electrode type with four types of dry electrodes. The subjects performed pre-arranged movements to provoke artifacts. The calculated signal-to-artifact ratios showed that the gel electrode was the best, followed by titanium, silver, stainless steel, and rubber^25^. In the present study, the stainless steel performed the best in stationary testing, followed by platinum, silver, conductive polymer, and conductive fabric. In the movement testing, platinum and stainless steel switched places, with the rest of the order remaining the same.

Testing dry electrode materials on human skin is challenging. Test benches with synthetic skin provide stable and repeatable testing conditions^76^ but do not fully model the anatomy and physiology of skin. It has been found that the results between the test bench and testing on skin show only moderate correlation^77^. Although the human skin is the correct environment for testing the electrodes for electrophysiological monitoring, the skin properties vary not only between individuals but also within individuals at different times. Another source of variance is the test method: the electrode locations, the way in which the electrodes are contacting the skin, the variance in the pressure of the electrodes on the skin, and the way in which motion artifacts are generated. These may all affect the results and generate significant variation between the studies, as is demonstrated by the results reviewed above.

Stainless steel appears to be a low-noise material by itself^78^ and on the skin, as found in the present and previous studies^14,23^. A wrist device featuring stainless steel dry electrodes for recording intermittent long-term ECG has recently been validated for atrial fibrillation detection^79^. In this application, the test subjects were stationary during the recordings, which averts the issue of a lowered SNR during movement. The majority of the dry electrode studies use flat electrodes—however, in our unpublished field tests with textile-integrated stainless steel dry electrodes, we have seen that a convex shape improves the electrodes’ robustness to motion artifacts compared to flat electrodes. Ödman has previously found in laboratory testing that more curved electrode surface is translated into higher skin potentials and lowered wear comfort^80^.

### Wearing comfort

In addition to the shape of an electrode, its material properties affect wearing comfort. In a recent review on dry electrode materials, Kim et al*.* discuss the wearing comfort and breathability—*i.e.,* the water vapor transmission rate ranging from 0.31 to 23 mg/cm^2^/h—of electrodes. The breathability of the electrode is important for the user’s comfort and wearability, and for preventing skin problems during long-term monitoring^28^. Wu et al*.* quantified the wearing comfort and signal quality of conductive fabric electrodes^56^. Low air resistance and high thermal conductivity allow the transfer of moisture and heat from the skin–electrode interface to the ambient air, improving the wearing comfort. A honeycomb knit was shown to have lower air resistance than a plain knit. Test subjects wearing large 100 mm × 100 mm electrodes manufactured from these knits gave the best wearing comfort score to the honeycomb despite it having the lowest thermal conductivity. They also gave the lowest score to the plain knit electrode which had the highest thermal conductivity. Therefore, in subjective wearing comfort, breathability, i.e., low air resistance, appears to be a more important factor than high thermal conductivity. We did not study wearing comfort, but the order of air resistance and thus wearing comfort could be derived from the electrode structures. The order was from highest to lowest air resistance: solid electrodes, conductive polymer, conductive fabric with a membrane, and conductive fabric without a membrane. In our current results, the conductive fabric without a moisture-retaining membrane showed the lowest performance in terms of SNR. These results are in line with the findings of Wu et al*.* However, the electrode size affects the wearing comfort. In large electrodes, the wearing comfort becomes a factor. In small electrodes, such as those used in our study, 3% of the area of the electrodes used by Wu et al*.*, any biocompatible material appeared to be well-tolerated and did not cause skin irritation.

Practical experience has also shown that signal quality and wearing comfort are often inversely related to each other. For example, an electrode with a large surface area that does not allow moisture to escape and is tightly fixed onto the skin with snug garments will produce higher-quality signals but will feel uncomfortable over an extended wearing time. In contrast, an electrode with a small surface area that allows the skin to breath and is integrated into loosely fitting garments is comfortable to wear but records artifact-filled signals. Therefore, optimization is needed when designing electrodes for wearables intended for long-term use by finding a balance between factors that influence signal quality and wearing comfort, such as electrode surface area, pressure, curvature, breathability, and heat transfer. Comprehensive and thorough understanding of the application is essential to tailor the optimization of the electrodes effectively for their intended use.

### Dry electrode and amplifier noise

The noise results of the used amplifier and electrodes show that the amplifier has a negligible influence on the overall noise of the measurement. A high-quality amplifier is a strict requirement for ECG recordings to avert contaminating the data with noise^40,45,74,81^.

The conductive polymer has a higher peak-to-peak noise voltage and total noise power than the metallic electrodes in the recorded 0.05–100 Hz band. This may be due to the higher internal resistance of the conductive polymer, 330 Ω for a pair and 165 Ω per electrode, than that of the metallic electrodes, from 0.050 Ω to 0.58 Ω per pair and from 0.025 Ω to 0.29 Ω per electrode, as shown by Kaappa et al.^82^. The input noise current of the amplifier passes through the resistance of the electrode where it is converted into noise voltage^45^. Even though there is a significant difference in the electrodes’ internal resistances, the skin–electrode interface impedance can be several megaohms, as seen in the current study. Therefore, the resistance of the electrode material itself has little significance to the whole measurement. The manufacturer cites an input noise current for Biopac MP35 and MP36 of 100 fA_rms_ / √Hz. Even with a 1 MΩ skin–electrode interface impedance, the noise voltage due to the input noise current is approximately 1 µV_rms_ and much less than the V_p-p_ results in Table 5. Therefore, the noise seen in the measurement is predominantly something else besides the intrinsic noise of the amplifier, electrodes, or skin–electrode interface. Upon visually inspecting the data, it was observed that there were no electromyographic bursts; however, interference from muscle tone might still be present. It should be noted that the inter-electrode distance in the present study was 120 mm as opposed to the 30 mm in the reference study^45^, allowing more biological noise signal sources to interfere with the measurement. Also, An et al*.* has showed with dry conductive fabric electrodes that the skin–electrode impedance varied in repeated measurements over ten days from approximately 140 kΩ to 470 kΩ due to electrode locations and the subject’s skin properties in the forearm, as well as approximately from 145 to 235 kΩ due to electrode pressure^27^. The noise is probably a combination of the above-mentioned factors, as well as of the electric activity of the muscles of the forearm below the electrodes, even though the test subjects were sitting down and at rest.

Maji and Burke assessed the noise in one type of gelled electrode and five types of dry electrodes, conductive fabric with different backings and one type of conductive silicone rubber^45^. They fitted a K/f + C amplitude spectral density model to their data, where f is frequency and K and C are coefficients. Here, their results were converted to power spectral densities and limited to the bandwidth of the present study. The results were several decades lower than expected, in the order of 10^–15^–10^–14^ V^2^/Hz. The electrode surface areas that Maji and Burke used were 200–700% larger than those used in the present study. Puurtinen et al*.* showed that textile electrode noise level increases as the electrode size decreases^83^. In the study by Huigen et al*.*, the noise of gelled electrodes was inversely proportional to the square root of the electrode surface area^57^. The surface area difference alone does not explain the discrepancy in the results and may be attributed to noise from the muscle activity, as noted above.

## Limitations

The present study has some limitations. The study could have benefitted from having a known electrode as a reference, such as a disposable Ag/AgCl electrode. However, such electrodes have a hydrogel layer or a wet gel sponge to moisturize the stratum corneum, providing a well-conductive connection and adhesive to fix the electrode onto the skin. These features make the electrode incomparable to the tested electrode types, which are intended for long-term wearable applications. Also, the properties of the wet Ag/AgCl electrode would have changed markedly due to dehydration during the 48-h test period, making comparisons with dry electrodes less meaningful. The application of a gel or replacement of the electrode would also not make for a fair comparison, as the goal was to study the electrodes’ long-term performance with no user interaction. However, including a wet Ag/AgCl would have provided a point of reference and, perhaps, a point in time when the dry electrodes’ signal quality surpasses that of the Ag/AgCl.

The users’ varying skin properties may affect the spread of the results. Tam and Webster report that measured skin potentials can have marked inter-subject variation. Even when using the same subject, the same sites, and the same amount of time for electrode stabilization, the skin potential differed by ± 10 mV between trials^17^. Sunaga et al*.* studied the dielectric properties of skin and found differences of > 20% between two subjects^68^. In the present study, six test subjects were assigned for each electrode type. Indeed, the spread of data in the results was high and, as can be seen from the range covered by the whiskers in Fig. 6. A large number of test subjects could have yielded distributions nearing the normal distribution and increased confidence in the results regarding the differences between the materials. However, it was challenging to find test subjects ready to commit to a 48-h study with the restrictions on exercise and skin care. Furthermore, these results showed clear SNR trends with the studied materials, which may persist even with a larger set of test subjects.

The electrode materials or the minimal skin abrasion of the electrode on skin did not cause skin irritation. According to the Prokerala and Fitzpatrick tests, none of the test subjects had dry or sensitive skin or high-Fitzpatrick skin types that would have been more prone to adverse effects from wearing the electrodes. However, the results do not necessarily exclude the possibility of irritation, as different factors, such as extremely sensitive skin, previous sensitization to the electrode materials, a large electrode area, or long exposure, may increase the risk.

## Conclusions

Cardiovascular diseases are the leading cause of death globally, causing approximately a third of all deaths. Electrocardiography is a valuable tool in diagnosing and monitoring cardiovascular diseases. Wearable electronics can record ambulatory ECG, which can be sent to health care professionals for review and decision making. For long-term use, it is important that the electrodes do not cause skin irritation, which can be seen with disposable wet electrodes. It is also important that the electrodes can provide as good a signal quality as possible during movement.

In this study, ECG measurement results on dry electrode materials for wearable applications were described. Signal-to-noise ratio calculations showed that solid dry electrode materials can measure higher-quality ECG than porous materials in both the short and the long term. Skin–electrode impedances were higher in porous than in solid electrodes. SNR and impedance are negatively associated with each other. Of the tested solid materials, stainless steel electrodes provided the highest-quality ECG in stationary tests. In the movement provocation test, platinum demonstrated the highest-quality ECG, followed by stainless steel.

## Data Availability

The data supporting the results reported in the article is available in IEEE Dataport^31^ and Mendeley Data^32^.
